# Comparison of clinical and radiological outcomes between microscope-assisted tubular unilateral laminectomy for bilateral decompression and bilateral laminotomy for crossover decompression in single-level lumbar spinal stenosis

**DOI:** 10.3389/fsurg.2026.1919696

**Published:** 2026-08-10

**Authors:** Abuduwahapu Aierken, Ajiguli Waisiding, Alimujiang Yusufu, Yuan Ma

**Affiliations:** The Sixth Affiliated Hospital of Xinjiang Medical University, Urumqi, Xinjiang Uygur Autonomous Region, China

**Keywords:** bilateral laminotomy for crossover decompression, dural sac cross-sectional area, facet joint preservation, lumbar spinal stenosis, minimally invasive decompression, unilateral laminectomy for bilateral decompression

## Abstract

**Objective:**

To compare the clinical and radiological outcomes of microscope-assisted unilateral laminectomy for bilateral decompression (ULBD) and bilateral laminotomy for crossover decompression (BLCD) in the treatment of single-level lumbar spinal stenosis (LSS).

**Methods:**

This retrospective comparative study included 65 patients with single-level LSS who underwent microscope-assisted tubular decompression between July 2023 and August 2024. Thirty-five patients underwent ULBD, and 30 underwent BLCD. The operative approach was selected in routine clinical practice without a predefined standardized anatomy-based allocation protocol. Baseline characteristics, operative time, and postoperative hospital stay were compared between the two groups. Clinical outcomes were assessed using visual analogue scale (VAS) scores for low back pain and leg pain and the Oswestry Disability Index (ODI) preoperatively and at postoperative follow-up time points. Radiological outcomes included changes in segmental lordosis and global lordosis, ipsilateral and contralateral facet joint preservation rates, postoperative dural sac cross-sectional area (DSCA), and DSCA change rate.

**Results:**

Baseline demographic and clinical characteristics were comparable between the two groups. The ULBD group had a significantly shorter operative time than the BLCD group (105.3 ± 9.5 min vs.128.7 ± 11.3 min, *P* < 0.001), whereas postoperative hospital stay did not differ significantly. VAS scores for low back pain and leg pain and ODI scores improved significantly after surgery in both groups at all follow-up time points compared with baseline (*P* < 0.05), with no significant between-group differences. Changes in segmental lordosis and global lordosis were not significantly different between groups. The ipsilateral facet joint preservation rate was significantly lower in the ULBD group than in the BLCD group (67.55% ± 4.72% vs. 89.23% ± 7.89%, *P* < 0.001), while the contralateral preservation rate showed no significant difference. Postoperative DSCA and DSCA change rate were significantly greater in the BLCD group than in the ULBD group (*P* < 0.001).

**Conclusion:**

Both microscope-assisted ULBD and BLCD provide effective symptom relief and functional improvement in patients with single-level LSS. ULBD is associated with a shorter operative time, whereas BLCD provides better ipsilateral facet joint preservation and a greater radiological decompression effect. BLCD may therefore offer potential advantages in preserving posterior bony structures and achieving more extensive spinal canal decompression, while maintaining comparable short-term clinical efficacy.

## 1 Introduction

Lumbar spinal stenosis (LSS) is one of the most prevalent degenerative conditions encountered in spinal surgery. Its pathogenesis is characterized by a reduction in the effective spinal canal volume resulting from intervertebral disc herniation, hypertrophy of the ligamentum flavum, facet joint arthropathy, and osteophyte formation, which in turn leads to neural compression and the emergence of corresponding clinical symptoms (1). With the accelerating aging of the global population, the prevalence of LSS has been steadily increasing, and it is currently estimated to affect approximately 103 million individuals worldwide (2, 3). For patients who fail to respond to conservative management and experience progressive neurological deterioration, surgical decompression remains the mainstay of treatment to alleviate neural impingement and restore neurological function (4).

Traditional open total laminectomy decompression, although effective in achieving thorough neural release, often compromises postoperative lumbar sagittal alignment owing to extensive resection of the posterior column structures, and may predispose to secondary instability in the long term (1, 2). In an effort to reduce surgical trauma while preserving spinal integrity, minimally invasive decompression techniques have been developed (5). In 1988, Young et al. first described the unilateral laminotomy for bilateral decompression (ULBD), a procedure that accomplishes contralateral decompression by traversing the base of the spinous process via a unilateral approach, thereby achieving adequate bilateral neural release while maintaining the spinous process, the contralateral lamina, and the majority of posterior column elements (6, 7). Subsequently, the technique of microscopic bilateral decompression (MBD) performed through a tubular retractor system has matured and has been consistently shown to effectively alleviate lumbosacral radicular symptoms, improve ambulatory capacity, and enhance quality of life in LSS patients (1, 2). Compared with conventional open surgery, MBD-ULBD offers distinct advantages, including less paraspinal muscle dissection, reduced intraoperative blood loss, shorter hospital stays, and accelerated postoperative recovery (8).

Alongside the development of ULBD, bilateral-crossing decompression has evolved as a distinct structure-preserving strategy for lumbar spinal stenosis. In 2015, Shin et al. (9) described bilateral decompression via microscopic tubular crossing laminotomy (MTCL), in which bilateral paramedian tubular corridors were used to decompress each lateral recess through a contralateral trajectory. Their early clinical series demonstrated substantial enlargement of the thecal-sac cross-sectional area, limited facet joint resection, and no radiographic instability during follow-up, establishing the feasibility of the bilateral-crossing concept under microscopic visualization (10). Subsequently, Lee et al. (11) translated this concept to unilateral biportal endoscopy as bilateral-contralateral decompression. Beyond describing the operative technique, these studies proposed an anatomy-based rationale for minimizing facet joint violation in selected vulnerable conditions, including stable low-grade degenerative spondylolisthesis, adjacent-segment stenosis after lumbar fusion, and upper-lumbar stenosis characterized by narrow laminae and vertically oriented facet joints (12). These reports suggest that the principal structural advantage of bilateral-crossing decompression is not merely the use of bilateral access corridors, but the ability to decompress both lateral recesses through contralateral working trajectories while limiting direct ipsilateral facetectomy.

Nevertheless, the available evidence remains limited and is derived predominantly from retrospective case series and technical reports. Moreover, direct comparative evidence between microscope-assisted tubular ULBD and microscope-assisted bilateral-crossing decompression remains scarce. A comprehensive set of outcome measures were assessed, including operative time, estimated blood loss, length of hospital stay, visual analogue scale (VAS) scores for pain, Oswestry Disability Index (ODI), complication rates, and radiological parameters, with the aim of providing evidence-based guidance for the optimal selection of minimally invasive surgical approaches in the management of lumbar spinal stenosis.

## 2 Materials and methods

### 2.1 General information

This was a retrospective comparative study. The study protocol was approved by the Medical Ethics Committee of Sixth Affiliated Hospital of Xinjiang Medical University (approval No. 20230628096), and all patients provided written informed consent and voluntarily participated in the study. Patients with LSS who underwent microscope-assisted tubular surgery in the Department of Spine Surgery at the Sixth Affiliated Hospital of Xinjiang Medical University from July 2023 to August 2024 were enrolled as research subjects.

All operations were performed by a single senior spine surgeon (Y.M.) with more than 10 years of experience in minimally invasive spinal surgery. The surgeon had completed more than 100 microscope-assisted tubular decompression procedures prior to the start of this study. The ULBD technique was introduced in our department in 2019, and BLCD was adopted in 2022. Both procedures were performed by the same surgical team using a standardized perioperative protocol.

### 2.2 Inclusion and exclusion criteria

**Inclusion criteria:** ① clinically and radiologically confirmed single-level LSS; ② failure of standard conservative treatment for more than 3 months; ③ neurogenic intermittent claudication as the main clinical manifestation; ④ clinical symptoms and signs consistent with imaging findings; ⑤ postoperative follow-up of at least 12 months with complete clinical and radiological data.

**Exclusion criteria:** ① previous surgery at the same level; ② concomitant ossification of the posterior longitudinal ligament or congenital developmental spinal stenosis; ③ associated disc herniation, lumbar spondylolisthesis, or scoliosis; ④ combined vertebral fracture, intraspinal infection, or tumor; ⑤ age <18 years; ⑥ severe underlying comorbidities precluding tolerance to surgery.

### 2.3 Selection of surgical approach

The choice between ULBD and BLCD was not based on a prospectively established or standardized anatomy-based allocation protocol. In routine clinical practice, the operative approach was selected on a case-by-case basis by the operating surgeon after reviewing the clinical manifestations and preoperative CT and MRI findings. However, no predefined quantitative thresholds regarding the severity or symmetry of lateral recess stenosis, laminar width, facet orientation, or other anatomically vulnerable characteristics were consistently applied or documented. Therefore, treatment allocation was nonrandom and may have been influenced by surgeon preference and other unmeasured clinical or anatomical factors.

### 2.4 Surgical procedure

#### 2.4.1 ULBD group

After general anesthesia, the patient was placed in the prone position. The target level was localized using a C-arm fluoroscope. A longitudinal incision of approximately 2 cm was made about 2 cm lateral to the spinous process. After sequential dilation, a Quadrant minimally invasive retractor (Medtronic, USA) was inserted, and its position was reconfirmed by fluoroscopy. After connecting the microscope, the soft tissue on the lamina surface was cleared with an electrocautery. A burr was used to remove part of the ipsilateral spinous process root, the inferior lamina margin, and the medial portion of the inferior articular process, exposing the upper and lower attachment points of the ligamentum flavum. A nerve dissector was used to separate adhesions between the ligamentum flavum and the dura mater, and the lateral part of the ipsilateral ligamentum flavum was resected under direct vision (with approximately one-third of the medial part preserved to protect the dura mater). Subsequently, a Kerrison rongeur was used to remove medial osteophytes of the superior articular process, enlarge the nerve root canal, expose the ipsilateral traversing nerve root and dura mater, which were then covered with saline-soaked cottonoid patties for protection. After completing ipsilateral decompression, the operating table was tilted about 15° to the contralateral side, and the retractor was angled contralaterally. The contralateral spinous process root and the medial-inferior edge of the lamina were burred to expose and resect the contralateral ligamentum flavum, and the contralateral nerve root canal and lateral recess were explored to ensure adequate decompression. After protecting the contralateral dura with cottonoid patties, the retractor was removed, a drainage strip was placed, and the incision was closed in layers.

#### 2.4.2 BLCD group

Patient positioning, anesthesia, and fluoroscopic localization were identical to those used in the ULBD group. To avoid ambiguity, the side on which the tubular retractor was currently positioned was defined as the approach side, whereas the opposite side undergoing decompression through the crossover trajectory was defined as the target side. In the first stage, a longitudinal paramedian incision of approximately 2 cm was made on the first approach side, and a Quadrant tubular retractor was introduced through sequential dilation. Under microscopic visualization, the interlaminar space and the lamina–spinous process junction were exposed. A limited laminotomy was performed to create a sublaminar crossover working corridor. At this stage, complete direct decompression of the lateral recess on the approach side was not performed. The operating table was then tilted approximately 15° toward the target side, namely, away from the active approach side. The tubular retractor and the microscopic visual axis were angled in the same direction. This maneuver aligned the working trajectory beneath the base of the spinous process and improved visualization of the contralateral lamina, lateral recess, and traversing nerve root. The medial portion of the spinous process base and the medial-inferior margin of the target-side lamina were undercut using a high-speed burr. The ligamentum flavum and, when necessary, medial osteophytes of the target-side superior articular process were removed until the target-side lateral recess and traversing nerve root were adequately decompressed. A saline-soaked cottonoid patty was placed over the medial margin of the decompressed dural sac as both a protective layer and an anatomical landmark. In the second stage, a corresponding paramedian incision was made on the opposite side, and a second tubular corridor was established. The same procedure was performed in a mirror-image fashion. The operating table was tilted approximately 15° toward the second target side, which was the side decompressed through the first approach corridor. The retractor and microscope were angled toward this target side, and crossover undercutting of the spinous process base and opposite lamina was performed. Decompression was continued until the previously placed cottonoid patty was visualized at the medial boundary of the first decompression field, confirming adequate overlap of the two decompression corridors and complete central decompression. Residual ligamentum flavum and osteophytes were removed, and both traversing nerve roots were inspected. Adequate decompression was confirmed by visualization of the freely pulsating dural sac and free passage of a nerve hook along both lateral recesses and traversing nerve roots. After meticulous hemostasis, drainage strips were placed as required, and both incisions were closed in layers.

#### 2.4.3 Postoperative management

All patients received a standardized perioperative management protocol. Routine prophylactic antibiotics were administered postoperatively, and the drainage strips were removed 24–48 h after surgery based on the drainage volume. From the first postoperative day, patients were instructed to perform ankle pump exercises and isometric quadriceps contractions in bed to prevent deep vein thrombosis of the lower extremities. On the 2nd to 3rd postoperative day, patients were encouraged to ambulate with the protection of a lumbar brace, which was worn for 4–6 weeks. After discharge, patients were guided to perform back muscle strengthening exercises, and were advised to avoid strenuous exercise and heavy physical labor within the first 3 months postoperatively. All patients received rehabilitation instructions according to a unified protocol, and there were no differences in postoperative management between the two groups.

### 2.5 Outcome measures

#### 2.5.1 Perioperative parameters

Operative time (from skin incision to wound closure) and postoperative hospital stay were recorded for both groups.

#### 2.5.2 Clinical efficacy evaluation

The VAS was used to assess low back pain and leg pain, respectively. The ODI was used to evaluate patients' daily functional ability. All scores were collected preoperatively, at 1 week, 1 month, 3 months, 6 months postoperatively, and at the final follow-up.

#### 2.5.3 Imaging measurement protocol

All radiological measurements were performed by two independent observers (A.A. and A.W.) who were blinded to the surgical procedure and clinical outcomes. Preoperative and postoperative images were evaluated in random order to minimize recall bias. For CT measurements of facet joint width, the axial slice at the mid-portion of the target disc level was selected. For DSCA measurements on MRI, the axial T2-weighted image at the most stenotic level (identified by the smallest preoperative dural sac area) was used. Each measurement was performed twice by each observer, and the average value was used for analysis. Inter-observer reliability was assessed using intraclass correlation coefficients (ICC), with ICC > 0.80 indicating good agreement.

#### 2.5.4 Radiological evaluation

All patients underwent lumbar anteroposterior and lateral X-ray, CT, and MRI examinations preoperatively and at the final follow-up.

**Figure 1:**
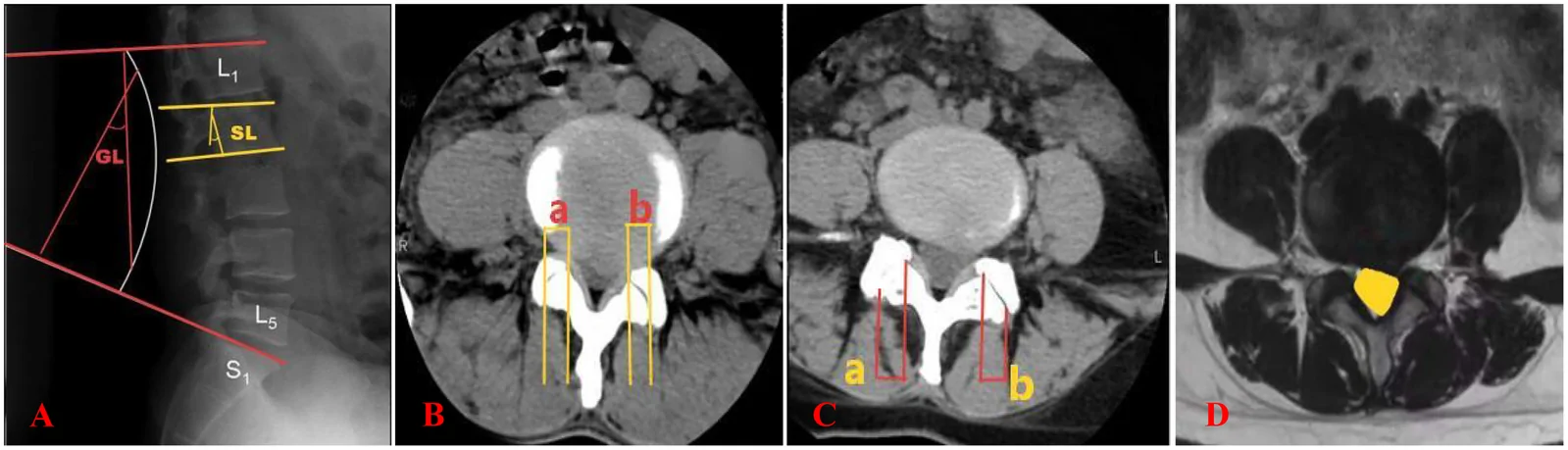
Schematic diagram of imaging measurements: **(A)** lateral lumbar radiograph measuring the whole-segment lordosis angle and segmental lordosis angle; **(B)** preoperative transverse CT measurement of articular process joint width a and b; **(C)** postoperative transverse CT measurement of articular process joint width a and b; **(D)** postoperative transverse MRI measurement of the cross-sectional area of the dural sac.

1. Assessment of lumbar sagittal alignment: Segmental lordosis (SL) and global lordosis (GL) at the target level were measured on lateral lumbar X-ray films preoperatively and at the final follow-up. The difference (postoperative value − preoperative value) was calculated; a smaller difference indicated better maintenance of postoperative lumbar sagittal alignment. We acknowledge that static lateral radiographs reflect sagittal alignment rather than dynamic instability; dynamic flexion-extension radiographs were not routinely obtained in all patients, which is a limitation of this study. (Figure 1A).
2. **Facet joint preservation rate**: On preoperative and postoperative axial CT images at the target level, the width of the bilateral facet joints was measured. The ipsilateral and contralateral facet preservation rates were calculated as the percentage of postoperative width relative to preoperative width (Figures 1B,1C).
3. **Dural sac cross-sectional area (DSCA)**: On axial T2-weighted MRI images at the most stenotic level, DSCA was manually delineated along the dural sac margin preoperatively and at the final follow-up (Figure 1D). The DSCA change rate was calculated as: (postoperative DSCA − preoperative DSCA)/preoperative DSCA × 100% (13).

### 2.6 Statistical analysis

Statistical analysis was performed using SPSS version 27.0. All continuous variables were first tested for normality using the Shapiro–Wilk test. Normally distributed data were expressed as mean ± standard deviation (x¯ ± s), and categorical data as numbers (percentages). Comparisons between the two groups for baseline data, perioperative parameters, and radiological parameters were performed using the independent-samples *t*-test for normally distributed variables and the Mann–Whitney *U*-test for non-normally distributed variables. Categorical variables were compared using the chi-square test. VAS scores and ODI scores at different time points (preoperatively, 1 week, 1 month, 3 months, 6 months, and at final follow-up) were compared using repeated-measures analysis of variance (ANOVA). When the sphericity assumption was violated, the Greenhouse–Geisser correction was applied. Post-hoc pairwise comparisons within groups between each postoperative time point and baseline were performed using Dunnett's method; between-group comparisons at the same time point were performed using the independent-samples *t*-test with Bonferroni correction for multiple comparisons. All tests were two-sided, and *P* < 0.05 was considered statistically significant.

## 3 Results

### 3.1 Patient selection process and general data

**Figure 2:**
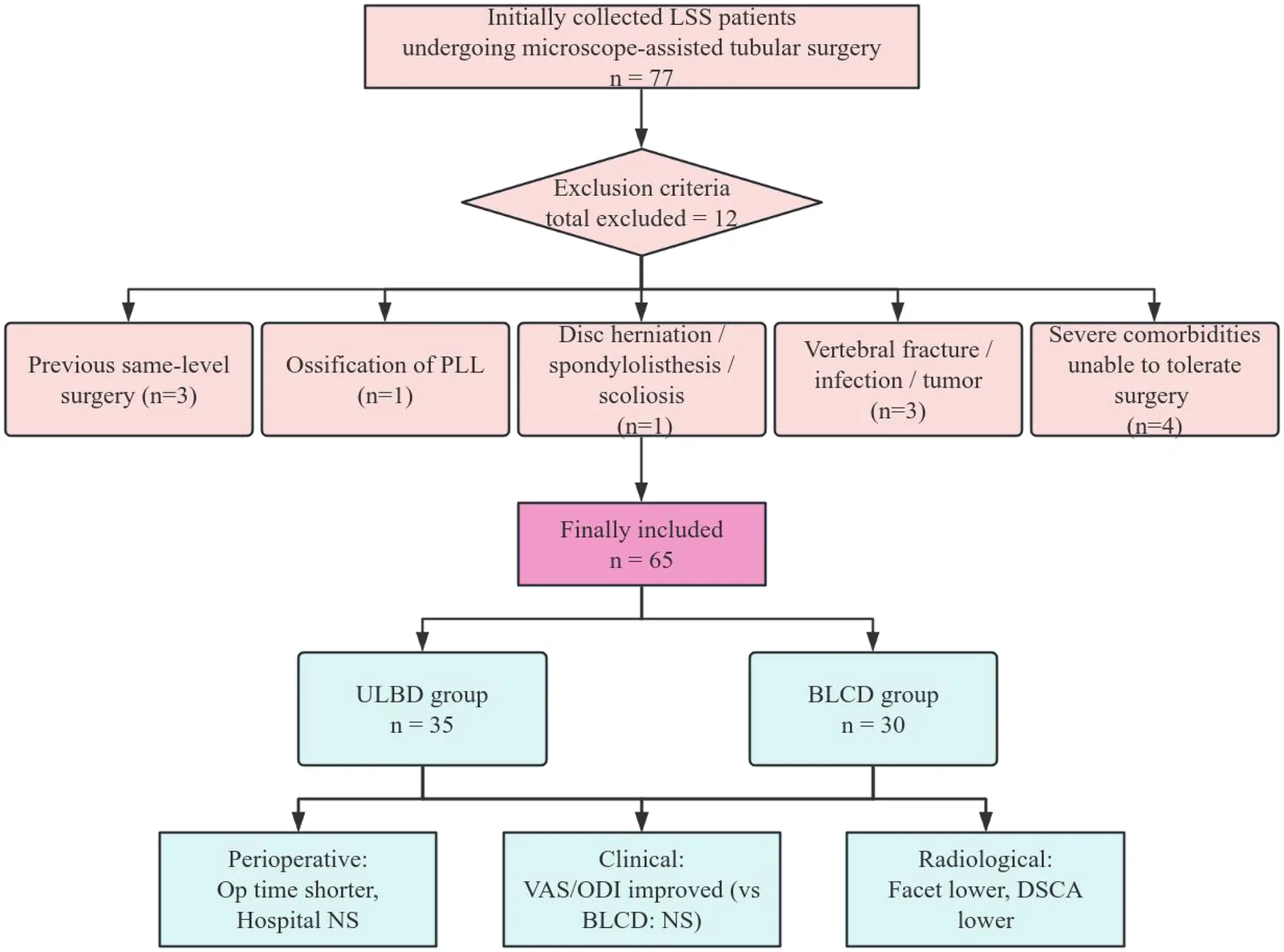
Flow chart of test grouping.

A total of 77 patients with LSS who underwent microscope-assisted tubular surgery at our hospital from July 2023 to August 2024 were initially screened. According to the exclusion criteria, 12 patients were excluded (3 with previous same-level surgery, 1 with ossification of PLL, 1 with disc herniation/spondylolisthesis/scoliosis, 3 with vertebral fracture/infection/tumor, and 4 with severe comorbidities unable to tolerate surgery). Finally, 65 patients were enrolled, including 35 in the ULBD group and 30 in the BLCD group (Figure 2). Baseline characteristics (sex, age, and involved segments) were comparable between the two groups (all *P* > 0.05).

### 3.2 Baseline data and perioperative parameters

No statistically significant differences were found between the two groups in sex distribution, age, level of stenosis, or preoperative comorbidities (hypertension, diabetes, and coronary heart disease) (all *P* > 0.05), indicating that the baseline data were comparable. In terms of perioperative parameters, the ULBD group had a significantly shorter operative time than the BLCD group (*P* < 0.05), whereas no significant difference was observed in postoperative hospital stay (*P* > 0.05). Estimated blood loss was significantly lower in the ULBD group than in the BLCD group (85.6 ± 32.4 mL vs. 112.3 ± 41.7 mL, *P* = 0.006). The overall complication rate was 5.7% (2/35) in the ULBD group and 10.0% (3/30) in the BLCD group, with no statistically significant difference (*P* = 0.662). All complications were managed conservatively without long-term sequelae. No reoperations or unplanned readmissions occurred in either group. as detailed in Table 1.

**Table 1:** Comparison of baseline data and perioperative parameters between the two groups (x¯ ± s).

Parameter	ULBD group (*n* = 35)	BLCD group (*n* = 30)	*t*/*χ*^2^	*P*-value
Sex (male/female, *n*)	18/17	16/14	0.023	0.879
Age (years)	62.4 ± 5.3	60.9 ± 6.8	0.987	0.327
Stenosis level (n)			0.199	0.905
L_3–4_	11	8		
L_4–5_	15	13		
L_5_S_1_	9	9		
Comorbidities (n, %)				
Hypertension	14 (40.0%)	11 (36.7%)	0.075	0.784
Diabetes	8 (22.9%)	7 (23.3%)	0.002	0.963
Coronary heart disease	5 (14.3%)	4 (13.3%)	0.012	0.914
Operative time (min)	105.3 ± 9.5	128.7 ± 11.3	−9.040	<0.001*
Postoperative hospital stay (d)	5.2 ± 0.8	5.4 ± 0.9	−0.941	0.351
Estimated blood loss (mL)^†^	85.6 ± 32.4	112.3 ± 41.7	−2.894	0.006*
Complications, *n* (%)^†^	2 (5.7%)	3 (10.0%)	0.424	0.662
- Dural tear	1 (2.9%)	1 (3.3%)	—	—
- Superficial infection	1 (2.9%)	1 (3.3%)	—	—
- Postoperative hematoma	0	1 (3.3%)	—	—
Reoperation	0	0	—	—
Unplanned readmission	0	0	—	—

* *P* < 0.05 indicates a statistically significant difference.

† These parameters were added after revision per reviewer request. All complications were managed conservatively without long-term sequelae.

### 3.3 Clinical efficacy evaluation

**Figure 3:**
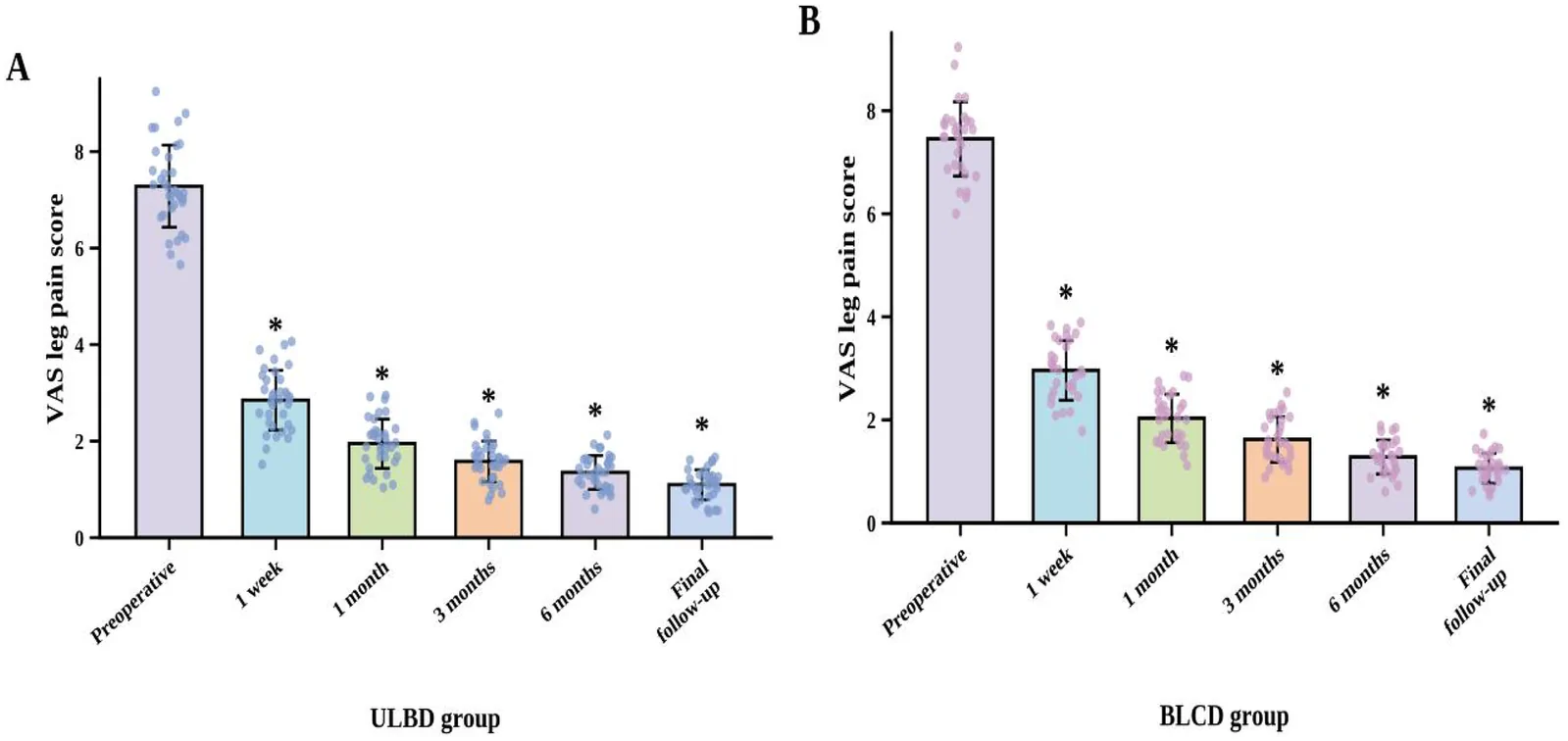
Comparison of preoperative and postoperative VAS scores for leg pain between the two groups. **(A)** ULBD group; **(B)** BLCD group.

**Figure 4:**
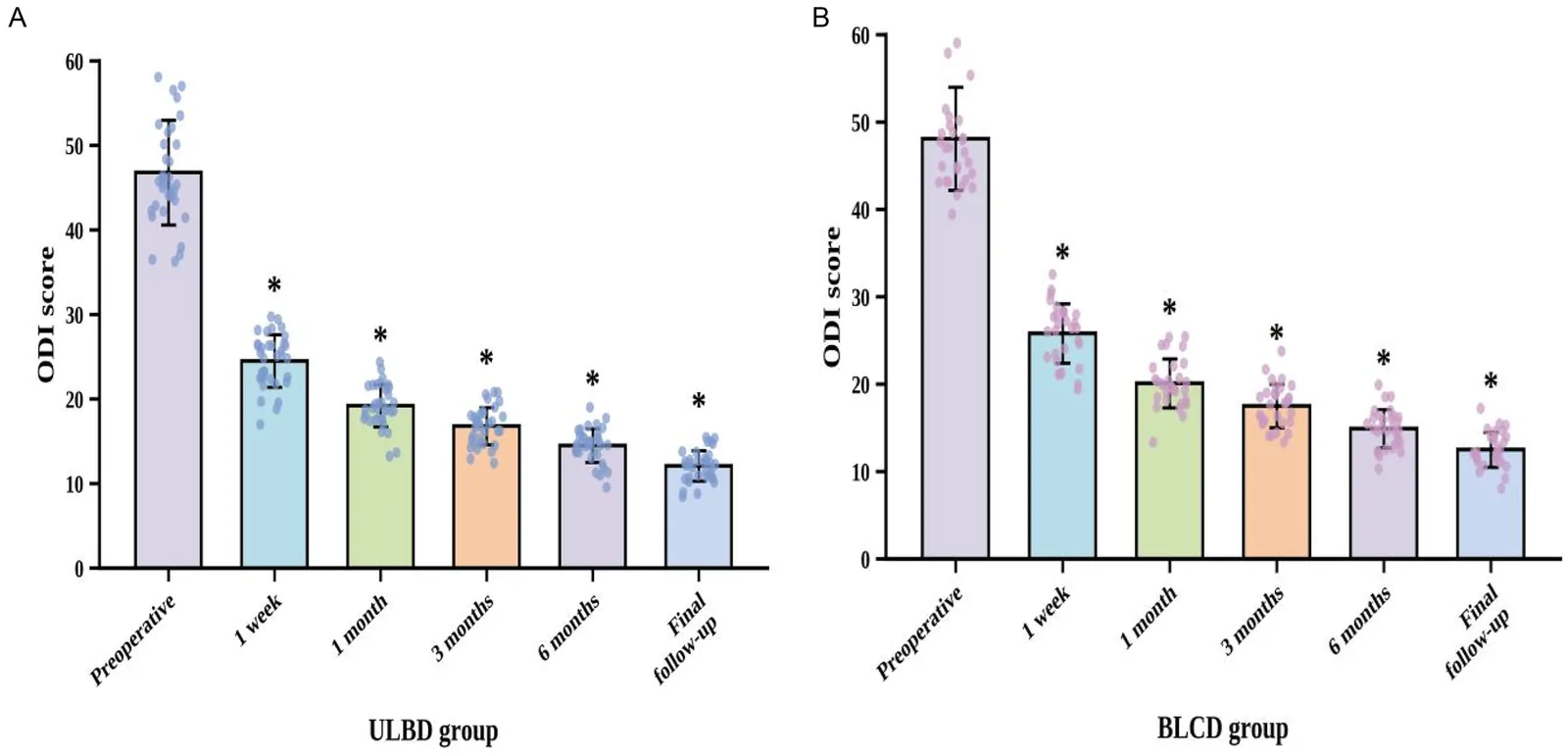
Comparison of preoperative and postoperative ODI scores between the two groups. **(A)** ULBD group; **(B)** BLCD group.

There were no statistically significant differences between the two groups in preoperative VAS scores for low back pain, VAS scores for leg pain, or ODI scores (all *P* > 0.05). At all postoperative follow-up time points (1 week, 1 month, 3 months, 6 months, and final follow-up), the VAS back pain scores, VAS leg pain scores, and ODI scores in both groups were significantly decreased compared with baseline (*P* < 0.05), indicating that both procedures effectively relieved pain and improved lumbar function. However, at any given follow-up time point, there were no statistically significant differences between the two groups in the above scores (*P* > 0.05), suggesting that ULBD and BLCD had comparable clinical efficacy in improving symptoms. Repeated-measures ANOVA showed a significant time effect (*P* < 0.001), but neither group effect nor group-by-time interaction was statistically significant (*P* > 0.05). The detailed data are shown in Tables 2–4 and Figure 3 (Repeated-measures ANOVA: time effect *F* = 945.78, *P* < 0.001; group effect *F* = 0.42, *P* = 0.519; interaction *F* = 0.18, *P* = 0.905.)- Figure 4 (ANOVA: time effect *F* = 768.34, *P* < 0.001; group effect *F* = 0.41, *P* = 0.522; interaction *F* = 0.34, *P* = 0.792).

**Table 2:** Comparison of preoperative and postoperative VAS scores for low back pain between the two groups (points, x¯ ± s).

Time point	ULBD group (*n* = 35)	BLCD group (*n* = 30)	*t*-value	*P*-value
Preoperative	7.05 ± 0.82	7.23 ± 0.75	−0.921	0.361
1 week postop	3.12 ± 0.54*	3.28 ± 0.61*	−1.115	0.269
1 month postop	2.15 ± 0.49*	2.32 ± 0.55*	−1.302	0.198
3 months postop	1.82 ± 0.44*	1.91 ± 0.48*	−0.785	0.436
6 months postop	1.45 ± 0.38*	1.52 ± 0.41*	−0.709	0.482
Final follow-up	1.28 ± 0.35*	1.35 ± 0.39*	−0.754	0.454

* Indicates *P* < 0.05 vs. baseline within the same group (Dunnett's method). Repeated-measures ANOVA: time effect F = 892.45, *P* < 0.001; group effect *F* = 0.57, *P* = 0.454; interaction *F* = 0.23, *P* = 0.873.

**Table 3:** Comparison of preoperative and postoperative VAS scores for leg pain between the two groups (points, x¯ ± s).

Time point	ULBD group (*n* = 35)	BLCD group (*n* = 30)	*t*-value	*P*-value
Preoperative	7.28 ± 0.85	7.45 ± 0.72	−0.858	0.394
1 week postop	2.85 ± 0.62*	2.96 ± 0.58*	−0.729	0.471
1 month postop	1.95 ± 0.51*	2.03 ± 0.47*	−0.650	0.519
3 months postop	1.58 ± 0.42*	1.62 ± 0.44*	−0.370	0.714
6 months postop	1.35 ± 0.35*	1.28 ± 0.33*	0.813	0.421
Final follow-up	1.10 ± 0.31*	1.06 ± 0.29*	0.535	0.604

* Indicates *P* < 0.05 vs. baseline within the same group (Dunnett's method). Repeated-measures.

**Table 4:** Comparison of preoperative and postoperative ODI scores between the two groups (points, x¯ ± s).

Time point	ULBD group (*n* = 35)	BLCD group (*n* = 30)	*t*-value	*P*-value
Preoperative	46.8 ± 6.2	48.1 ± 5.9	−0.861	0.392
1 week postop	24.5 ± 3.1*	25.8 ± 3.4*	−1.604	0.115
1 month postop	19.2 ± 2.5*	20.1 ± 2.8*	−1.362	0.178
3 months postop	16.8 ± 2.2*	17.5 ± 2.5*	−1.191	0.237
6 months postop	14.5 ± 2.0*	14.9 ± 2.2*	−0.767	0.447
Final follow-up	12.1 ± 1.8*	12.5 ± 2.0*	−0.838	0.404

* Indicates *P* < 0.05 vs. baseline within the same group (Dunnett's method). Repeated-measures.

### 3.4 Radiological evaluation

**Figure 5:**
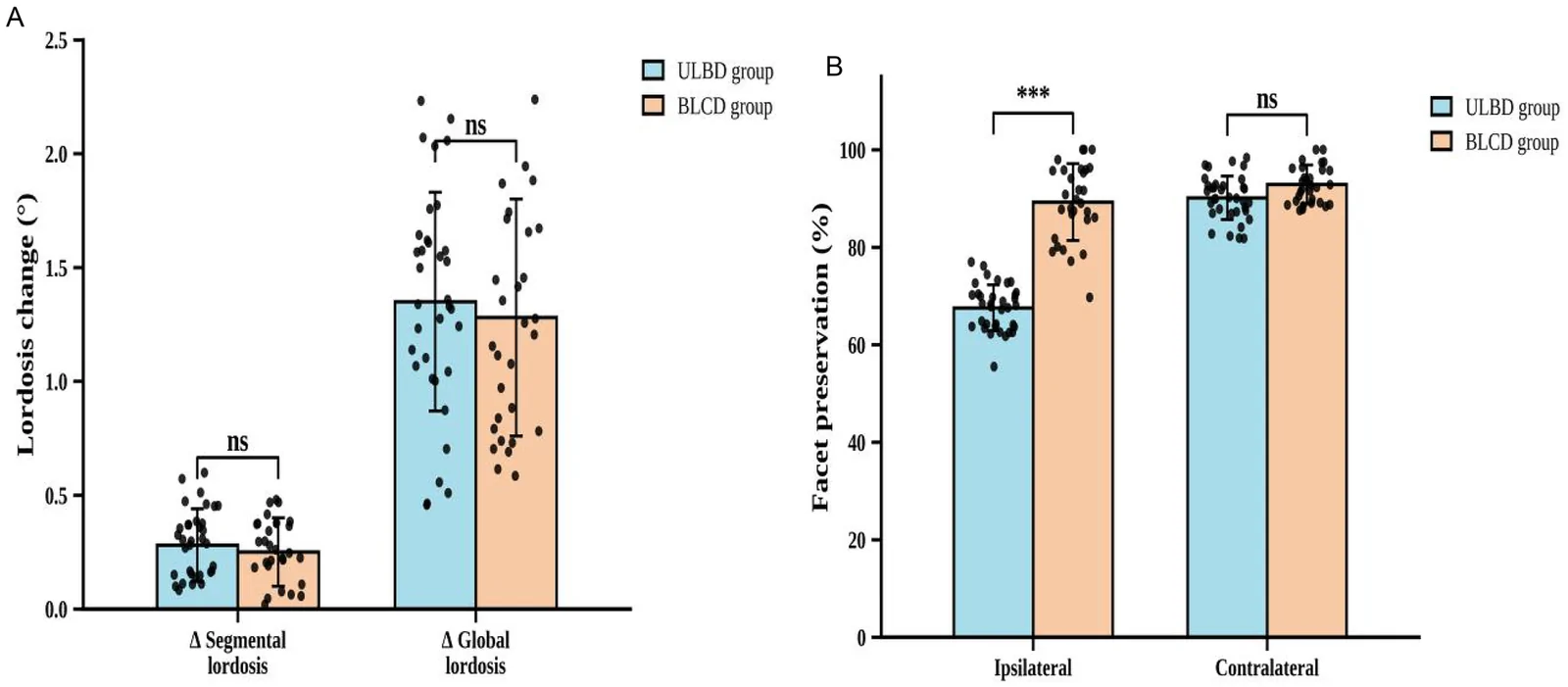
Comparison of changes in lumbar lordosis and facet joint preservation rates between the two groups. **(A)** Changes in segmental and global lordosis; **(B)** ipsilateral and contralateral facet preservation rates.

**Figure 6:**
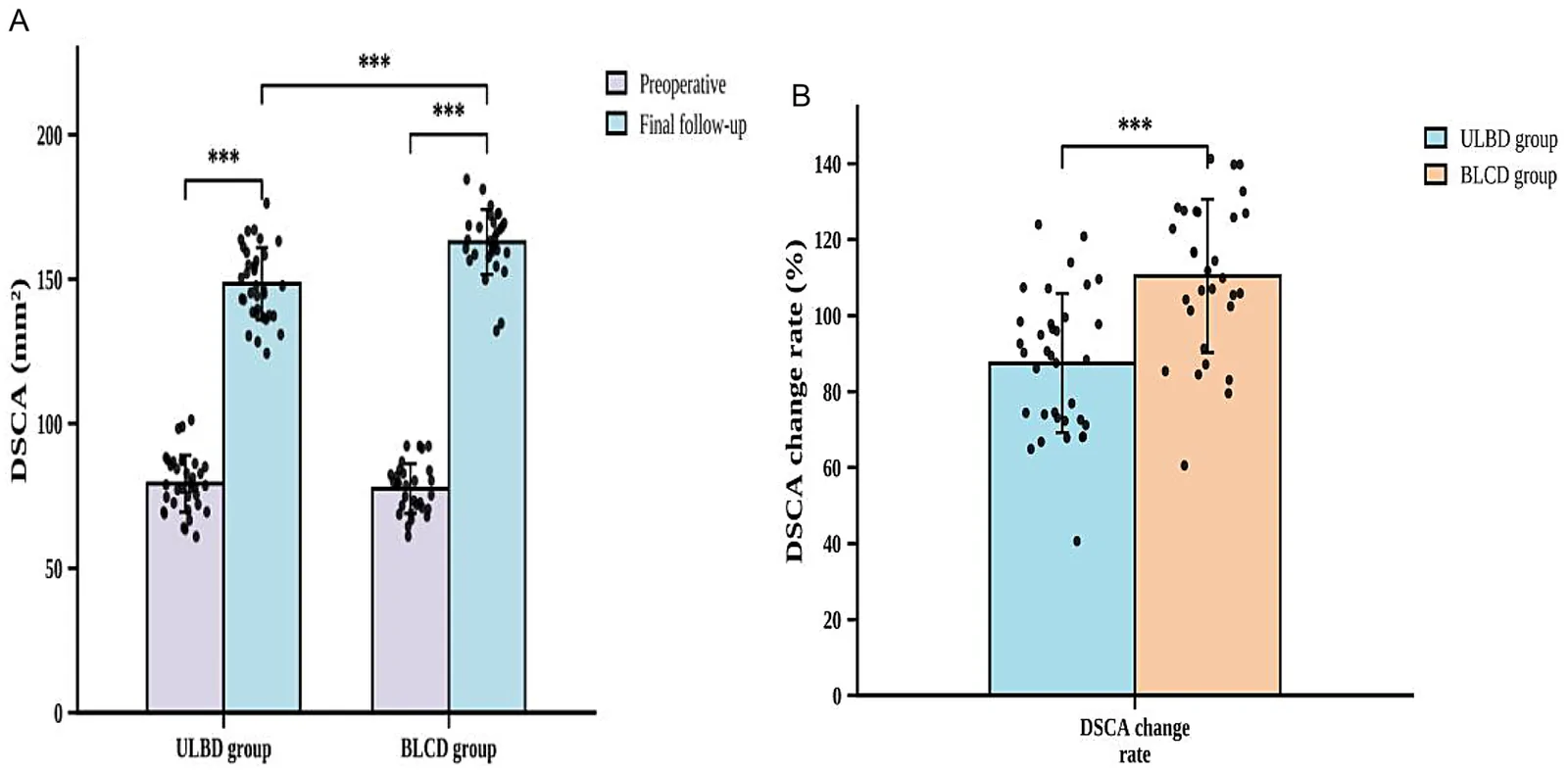
Comparison of dural sac cross-sectional area (DSCA) between the two groups. **(A)** Preoperative and postoperative DSCA; **(B)** DSCA change rate.

At the final follow-up, the differences (postoperative − preoperative) in segmental lordosis and global lordosis were not significantly different between the two groups (*P* > 0.05), indicating that both surgical techniques effectively maintained lumbar sagittal alignment during the follow-up period. The ipsilateral facet preservation rate in Group A (ULBD) was significantly lower than that in Group B (BLCD) (67.55 ± 4.72% vs. 89.23 ± 7.89%, *P* < 0.001), suggesting that ULBD involved a greater extent of bone resection at the ipsilateral facet joint. The contralateral facet preservation rate was significantly higher in the BLCD group than in the ULBD group (90.12 ± 4.48% vs. 92.85 ± 3.97%, *P* = 0.013), as shown in Table 5. Postoperative DSCA in both groups was significantly increased compared with baseline (*P* < 0.001), but the absolute postoperative DSCA and the DSCA change rate in Group B were significantly greater than those in Group A (*P* < 0.001), indicating that BLCD provided more extensive spinal canal decompression, as detailed in Table 6 and Figures 5, 6.

**Table 5:** Comparison of lumbar lordosis and facet joint preservation rates between the two groups (x ± s).

Parameter	ULBD group (*n* = 35)	BLCD group (*n* = 30)	*t*-value	*P*-value
Δ Segmental lordosis (°)	0.28 ± 0.16	0.25 ± 0.15	0.772	0.445
Δ Global lordosis (°)	1.35 ± 0.48	1.28 ± 0.52	0.571	0.574
Ipsilateral facet preservation (%)	67.55 ± 4.72	89.23 ± 7.89	−13.58	<0.001*
Contralateral facet preservation (%)	90.12 ± 4.48	92.85 ± 3.97	−2.573	0.013*

* Indicates *P* < 0.05, statistically significant difference. Δ values = final follow-up value minus preoperative value.

**Table 6:** Comparison of DSCA before and after surgery between the two groups (mm^2^, x¯ ± s).

Parameter	ULBD group (*n* = 35)	BLCD group (*n* = 30)	*t*-value	*P*-value
Preoperative DSCA	79.15 ± 9.82	77.43 ± 8.56	0.751	0.457
Final follow-up DSCA	148.36 ± 12.45*	162.78 ± 11.23*#	−4.889	<0.001*
DSCA change rate (%)	87.45 ± 18.32	110.42 ± 20.15#	−4.775	<0.001*

* Indicates *P* < 0.05 vs. baseline within the same group (paired *t*-test).

# Indicates between-group comparison, *P* < 0.05 (independent-samples *t*-test). DSCA change rate = (final DSCA − preoperative DSCA)/preoperative DSCA × 100%.

## 4 Discussion

Lumbar spinal stenosis is a highly prevalent degenerative spinal disorder, predominantly observed in middle-aged and elderly populations. The fundamental pathoanatomy involves narrowing of the lumbar spinal canal and lateral recesses due to hypertrophy of the ligamentum flavum, vertebral osteophytosis, and degenerative disc protrusion, which consequently compresses the nerve roots and dural sac, manifesting as lower extremity numbness, pain, and neurogenic claudication (14). For patients who are refractory to conservative management and exhibit persistently worsening symptoms, surgical decompression of the spinal canal remains the definitive curative intervention of choice. Traditional open total laminectomy decompression achieves radical neural release; however, it is fraught with substantial drawbacks, including extensive paraspinal muscle dissection, severe disruption of the posterior column structures, considerable intraoperative blood loss, and protracted postoperative recovery (15, 16). Moreover, it predisposes patients to long-term complications such as spinal instability and chronic low back pain, thereby posing considerable clinical limitations. With the advent of minimally invasive spinal techniques, microscope-assisted tubular decompression—characterized by superior visualization, reduced surgical trauma, and enhanced precision—has progressively supplanted conventional open surgery and become the mainstream therapeutic approach for lumbar spinal stenosis. Currently, the principal minimally invasive surgical modalities are ULBD and BLCD (17). Both techniques are capable of achieving adequate canal decompression while preserving the osseoligamentous integrity of the spine. Specifically, microscope-assisted ULBD requires only a unilateral surgical corridor, which minimizes soft tissue injury to the greatest extent, yet demands considerable technical proficiency from the surgeon (18). Conversely, BLCD affords more extensive visualization and facilitates more symmetrical bilateral nerve root decompression, although it carries a marginally higher risk of soft tissue trauma. To date, both domestic and international investigations have largely focused on independently validating the clinical efficacy of each procedure; systematic comparative studies that directly address disparities in surgical invasiveness, functional outcomes, complication rates, and long-term spinal stability between the two techniques remain conspicuously scarce, and a unified consensus regarding optimal surgical indication has yet to be established.

The present findings should be interpreted within the evolution of the bilateral-crossing decompression concept. Shin et al. demonstrated that microscopic tubular crossing laminotomy could provide bilateral neural decompression while limiting facet joint and multifidus injury (9). More recently, Lee et al. (11, 19) extended the bilateral-contralateral approach to endoscopic surgery and emphasized its potential structural value in anatomically vulnerable conditions in which direct ipsilateral decompression may require greater facet resection. The significantly higher ipsilateral facet preservation rate observed in our BLCD group is anatomically consistent with these previous studies because the crossing trajectory permits access to the lateral recess while reducing the need for direct medial facetectomy on the approach side. Similarly, the greater postoperative DSCA and DSCA change rate in the BLCD group support the ability of two independent crossing corridors to provide direct and relatively symmetrical bilateral decompression. Our study showed that the operative time in the ULBD group was significantly shorter than that in the BLCD group (105.3 ± 9.5 min vs. 128.7 ± 11.3 min, *P* < 0.001), a difference that carries clear clinical relevance. ULBD requires only a single surgical corridor to accomplish bilateral decompression, thereby avoiding the additional steps of secondary incision positioning, retractor placement, and contralateral soft-tissue dissection inherent to BLCD, which significantly shortens the operative duration. Hermansen et al. (20) conducted the NORDSTEN-SST randomized controlled trial, which included 437 patients with lumbar spinal stenosis and compared three midline-preserving decompression techniques-UL, BL, and spinous process osteotomy (SPO)-at 5year follow-up. They found no significant differences among the three techniques in ODI improvement or subsequent surgery rates. This large prospective study provides important external validity for our findings: although the bilateral approach does not confer an advantage in operative time, its long-term clinical efficacy is comparable to that of the unilateral approach, consistent with the conclusions of our short-term follow-up.

In terms of functional recovery, both groups showed significant improvements in VAS scores for low back pain and leg pain, as well as ODI scores, at all postoperative time points compared with baseline (*P* < 0.05), but no statistically significant differences were observed between the two groups at any time point. This suggests that, despite the different surgical approaches and techniques, both ULBD and BLCD effectively relieve neural compression, improve clinical symptoms, and restore lumbar function, with equivalent efficacy. Zhang et al. (21) compared the short-term effects of lumbar endoscopic Delta large-channel and microscopic tubular Quadrantchannel unilateral laminotomy with bilateral decompression in elderly patients with LSS, and similarly found no significant differences in postoperative VAS and ODI scores between the two modalities. He et al. (22) compared biportal endoscopic ULBD with uniportal endoscopic ULBD for LSS and also reported no statistically significant differences in ODI, VAS, or modified MacNab criteria between groups. These studies further corroborate our finding that, provided adequate neural decompression is achieved, clinical outcomes can be satisfactory regardless of the minimally invasive access route.

We evaluated lumbar sagittal alignment by measuring the changes (final follow-up minus preoperative) in segmental lordosis and global lordosis. There were no significant differences between the two groups in either segmental lordosis change (0.28° ± 0.16° vs. 0.25° ± 0.15°, *P* = 0.445) or global lordosis change, indicating that both techniques effectively maintain lumbar sagittal alignment. Previous studies have also confirmed that ULBD preserves lumbar lordosis and motor function within a 2-year follow-up, with no significant adverse effects on sagittal or coronal stability (23).

With respect to facet joint preservation, the two groups showed a marked difference. The ipsilateral facet preservation rate in the ULBD group was significantly lower than that in the BLCD group, whereas the contralateral preservation rate did not differ significantly between groups. This result reflects the fundamental difference in the extent of bony resection between the two techniques—ULBD requires greater resection of the ipsilateral medial facet joint and inferior lamina margin to obtain the working angle and space needed for contralateral decompression. The degree of ipsilateral facet resection is an important factor affecting postoperative lumbar sagittal alignment. Nonetheless, despite the lower ipsilateral facet preservation rate in the ULBD group, there was no significant difference in lumbar lordosis maintenance between groups, suggesting that within the range of single-level decompression, partial ipsilateral facet resection may not reach the threshold that influences sagittal stability. Previous studies have also indicated that although the facet preservation rate in minimally invasive endoscopic decompression groups is lower than that in open surgery groups, this lower rate does not translate into a higher incidence of postoperative instability (24). This finding has important clinical implications—surgeons should strive to preserve facet structures as much as possible while ensuring adequate decompression, but need not compromise the thoroughness of decompression out of excessive concern for stability.

Dural sac cross-sectional area (DSCA) is an objective radiological indicator for evaluating the degree of spinal canal decompression. Our study showed that postoperative DSCA increased significantly in both groups compared with baseline (ULBD: from 79.15 ± 9.82 mm^2^ to 148.36 ± 12.45 mm^2^; BLCD: from 77.43 ± 8.56 mm^2^ to 162.78 ± 11.23 mm^2^; within-group comparisons *P* < 0.001), but the postoperative DSCA and the DSCA change rate in the BLCD group were significantly greater than those in the ULBD group (*P* < 0.001). The greater postoperative DSCA observed after BLCD may be explained by its bilateral and complementary working trajectories. In ULBD, contralateral decompression is performed through a single unilateral corridor, and the extent of sublaminar undercutting and contralateral flavectomy may be constrained by the oblique viewing angle and limited instrument mobility. In contrast, BLCD provides two independent crossover corridors, allowing the medial lamina, ligamentum flavum, and lateral recess on each side to be addressed under a more favorable and relatively symmetrical visual axis. The overlap of the two decompression fields beneath the spinous process may also permit more complete removal of residual central and sublaminar compression. These anatomical and technical features may collectively account for the greater dural sac expansion observed in the BLCD group. However, greater DSCA should be interpreted as a radiological difference in decompression extent rather than evidence of superior clinical efficacy. The anatomical basis for this difference lies in the fact that BLCD, through two independent decompression channels, allows direct exposure and management of bilateral lateral recesses and nerve root canals, enabling the surgeon to remove bilateral compressive lesions more thoroughly under direct vision. In contrast, the contralateral decompression in ULBD is an “over-the-top” maneuver, limited by the working angle and visual field of a single channel, which may restrict the completeness of contralateral decompression to some extent. He et al. (22) also found that biportal endoscopic ULBD achieved a greater increase in postoperative DSCA and a larger contralateral facet resection angle compared with uniportal endoscopic ULBD. Marie-Hardy et al. (25) through quantitative MRI analysis, reported that DSCA increased significantly from 70.53 mm^2^ preoperatively to 172.2 mm^2^ postoperatively after minimally invasive decompression. These studies confirm the effectiveness of minimally invasive techniques in expanding the dural sac, while also suggesting that different approaches may yield quantitative differences in decompression extent.

However, the between-group difference in DSCA expansion did not translate into a difference in clinical efficacy. This seemingly paradoxical observation suggests a possible “threshold effect” in functional recovery—once decompression reaches a certain level and neural compression is sufficiently relieved, additional radiological expansion may not confer proportional clinical benefit. Studies on uniportal full-endoscopic ULBD have also found that functional outcomes are not significantly correlated with the extent of radiological decompression, as long as adequate exposure of the dural sac boundary is ensured (23). Therefore, when selecting a surgical technique, the surgeon should balance the completeness of decompression against surgical trauma, rather than pursuing maximal radiological expansion alone.

## 5 Limitations of the study

This study has several limitations. First, its retrospective design carries inherent selection bias; although the baseline characteristics of the two groups showed no statistical differences, the possibility of unmeasured confounding cannot be entirely excluded. Second, the relatively small sample size (35 in the ULBD group and 30 in the BLCD group) may limit the statistical power for some secondary outcome measures. Third, the follow-up duration was only 12 months, which is insufficient to evaluate long-term outcomes such as lumbar sagittal alignment and adjacent segment degeneration. Fourth, this study only included patients with single-level LSS, and the generalizability of our conclusions to multi-level stenosis requires further validation. Fifth, no predefined anatomy-based criteria were used to determine whether a patient should undergo BLCD rather than ULBD; the choice of surgical approach was made by the surgeon based on routine clinical assessment without a standardized quantitative protocol. Consequently, the present findings should be interpreted as associations within a retrospectively selected cohort and should not be considered evidence establishing specific anatomical indications for BLCD. Prospective studies using explicit anatomy-based selection criteria or randomized allocation are required. Sixth, postoperative lumbar sagittal alignment was assessed using static lateral radiographs rather than dynamic flexion-extension radiographs. Changes in segmental and global lordosis on static radiographs primarily reflect sagittal alignment and should not be interpreted as definitive evidence of lumbar stability. The absence of dynamic imaging represents a limitation of this study, and future studies incorporating flexion-extension radiographs are warranted to evaluate postoperative instability more accurately.

## 6 Summary

In summary, both microscope-assisted tubular ULBD and BLCD achieve satisfactory clinical outcomes and maintain lumbar sagittal alignment in the treatment of single-level lumbar spinal stenosis. ULBD offers a significant advantage in operative time, whereas BLCD demonstrates superior ipsilateral facet joint preservation and dural sac area expansion. Each technique has its own characteristics, and clinical selection should take into account the patient's specific anatomical conditions, the surgeon's technical proficiency, and the surgical objectives—ULBD is an ideal choice for patients in whom shorter operative time and less soft-tissue trauma are prioritized, while BLCD may be more advantageous for patients requiring more extensive decompression, especially those with severe bilateral lateral recess stenosis. Both techniques are safe and effective minimally invasive treatment options for lumbar spinal stenosis.

## Data Availability

The raw data supporting the conclusions of this article will be made available by the authors, without undue reservation.
